# Amplicon Sequencing of Rock-Inhabiting Microbial Communities from Joshua Tree National Park, USA

**DOI:** 10.1128/MRA.00494-21

**Published:** 2021-06-24

**Authors:** Claudia Coleine, Laura Selbmann, Nuttapon Pombubpa, Jason E. Stajich

**Affiliations:** a Department of Ecological and Biological Sciences, University of Tuscia, Viterbo, Italy; b Italian Antarctic National Museum (MNA), Mycological Section, Genoa, Italy; c Department of Microbiology and Plant Pathology, University of California, Riverside, Riverside, California, USA; d Institute of Integrative Genome Biology, University of California, Riverside, Riverside, California, USA; Vanderbilt University

## Abstract

Endolithic microorganisms have been reported to date in hot and cold drylands worldwide, where they represent the prevailing life forms ensuring ecosystem functionality, playing a paramount role in global biogeochemical processes. We report here an amplicon sequencing characterization of rocks collected from Joshua Tree National Park (JTNP), USA.

## ANNOUNCEMENT

Understanding the microbial community structure and diversity in dryland regions is of paramount importance in both microbial ecology and evolution. Under these conditions, where most life-forms cannot survive, rocks represent the main refuge for life. Indeed, the endolithic habitat provides thermal buffering, physical stability, and protection against UV and solar radiation, ensuring the ecosystem functionality and creating positive feedback for further colonization (1). Although endoliths are ubiquitous in drylands worldwide, including the hyperarid drylands, from Atacama Desert to the Antarctic Dry Valleys (for examples, see references 2 to 8), a comprehensive assessment of worldwide rock diversity remains insufficiently studied, and comparisons between geographic locations and different climatic conditions are still missing (9). The present study, to our knowledge, represents one of the few available amplicon sequencing reports within the hot desert of Joshua Tree National Park (JTNP) in California (for an example, see reference 10).

Ten rock samples were collected from three different sites in the JTNP (GPS, 34.10, −115.45; the elevation for all sites is between 1,040 and 1,340 m); the presence of endolithic colonization was assessed by direct observation *in situ*. Rocks were collected under sterile conditions using a geologic hammer and a chisel, placed in sterile bags, transported in a cooler with ice, and stored at −80°C at the University of California, Riverside (CA), until downstream analysis. DNA extraction was performed using 0.5 g of powdered rock with a PowerSoil DNA extraction kit (MoBio Laboratories, Carlsbad, CA, USA).

The fungal internal transcribed sequence 1 (ITS1) region was amplified using the primers ITS1F and ITS2 (11), while the bacterial 16S rRNA V4 region was amplified using 515F and 806R (12), according to the Earth Microbiome Project protocols (https://earthmicrobiome.org/protocols-and-standards/). Two replicates were amplified for each sample. PCRs were carried out with a total volume of 25 μl, containing 1 μl of each primer, 12.5 μl of *Taq* DNA polymerase (Thermo Fisher Scientific, Waltham, MA, USA), 9.5 μl of nuclease-free water (Sigma-Aldrich, St. Louis, MO, USA), and 5 ng of DNA template using an automated thermal cycler (Bio-Rad, Hercules, CA, USA). The ITS1 locus was amplified following an initial denaturation at 94°C for 1 min and 35 cycles at 94°C for 30 s, annealing at 52°C for 30 s, and extension at 68°C for 90 s, followed by a final extension at 68°C for 7 min. The PCR for the V4 region followed a protocol of an initial denaturation at 94°C for 3 min, 35 cycles at 94°C for 45 s, annealing at 50°C for 1 min, and extension at 72°C for 90 s, followed by a final extension at 72°C for 10 min. The amplicons were quantified using the Qubit double-stranded DNA (dsDNA) high-sensitivity (HS) assay kit (Life Technologies, USA), purified using the Qiagen PCR cleanup kit (Macherey-Nagel, Hoerdt, France), and then pooled to produce an equimolar mixture. Sequencing was performed at the Institute for Integrative Genome Biology (IIGB; https://iigb.ucr.edu/), University of California, Riverside (USA), with the MiSeq reagent kit v3 on the Illumina MiSeq platform (2 × 300-bp paired-end format).

The ITS1 and 16S rRNA data sets were processed separately using the AMPtk v1.4.3 tool (https://github.com/nextgenusfs/amptk) (13), which removed barcodes and primers, removed chimeras, denoised sequences, and eliminated sequences of <250 bp using default parameters. Amplicon sequence variants (ASVs) were generated using DADA2 v1.16.0 (14); singletons and rare taxa (ASVs represented by ≤5 reads) were discarded as recommended by Lindahl et al. (15). Finally, AMPtk was used for taxonomic identification by a “hybrid” taxonomy assignment, which calculates a consensus last common ancestor (LCA) taxonomy based on the results of a global alignment using the USEARCH v9.2.64 (16), UTAX, and SINTAX databases.

The fungal ITS resulted in 960,527 quality-filtered reads clustered in 869 valid ASVs, while a total of 1,059,591 valid output reads were obtained for 16S rRNA, resulting in 3,221 ASVs. The majority of the identified ITS sequences recovered from all samples belonged to the phylum Ascomycota, followed by Basidiomycota, while *Actinobacteria* and *Proteobacteria* predominated in the 16S rRNA data set.

### Data availability.

The bacterial 16S rRNA and fungal ITS1 data sets generated and analyzed in the current study are available in the NCBI Sequence Read Archive (SRA) under BioProject accession number PRJNA706555 and in the Zenodo repository (https://doi.org/10.5281/zenodo.3960774).

## References

[1] Wierzchos J, DiRuggiero J, Vítek P, Artieda O, Souza-Egipsy V, Škaloud P, Tisza M, Davila AF, Vílchez C, Garbayo I, Ascaso C. 2015. Adaptation strategies of endolithic chlorophototrophs to survive the hyperarid and extreme solar radiation environment of the Atacama Desert. Front Microbiol 6:934. doi:10.3389/fmicb.2015.00934. 26441871 PMC4564735

[2] Pointing SB, Belnap J. 2012. Microbial colonization and controls in dryland systems. Nat Rev Microbiol 10:551–562. doi:10.1038/nrmicro2831. 22772903

[3] Meslier V, Casero MC, Dailey M, Wierzchos J, Ascaso C, Artieda O, McCullough PR, DiRuggiero J. 2018. Fundamental drivers for endolithic microbial community assemblies in the hyperarid Atacama Desert. Environ Microbiol 20:1765–1781. doi:10.1111/1462-2920.14106. 29573365

[4] Selbmann L, Isola D, Egidi E, Zucconi L, Gueidan C, de Hoog GS, Onofri S. 2014. Mountain tips as reservoirs for new rock-fungal entities: *Saxomyces* gen. nov. and four new species from the Alps. Fungal Divers 65:167–182. doi:10.1007/s13225-013-0234-9.

[5] Friedmann EI. 1982. Endolithic microorganisms in the Antarctic cold desert. Science 215:1045–1053. doi:10.1126/science.215.4536.1045. 17771821

[6] Cary SC, McDonald IR, Barrett JE, Cowan DA. 2010. On the rocks: the microbiology of Antarctic Dry Valley soils. Nat Rev Microbiol 8:129–138. doi:10.1038/nrmicro2281. 20075927

[7] Qu EB, Omelon CR, Oren A, Meslier V, Cowan DA, Maggs-Kölling G, DiRuggiero J. 2020. Trophic selective pressures organize the composition of endolithic microbial communities from global deserts. Front Microbiol 10:2952. doi:10.3389/fmicb.2019.02952. 31969867 PMC6960110

[8] Choe Y-H, Kim M, Lee YK. 2021. Distinct microbial communities in adjacent rock and soil substrates on a high Arctic polar desert. Front Microbiol 11:607396. doi:10.3389/fmicb.2020.607396. 33488547 PMC7819959

[9] Coleine C, Stajich JE, de Los Ríos A, Selbmann L. 2021. Beyond the extremes: rocks as ultimate refuge for fungi in drylands. Mycologia 113:108–133. doi:10.1080/00275514.2020.1816761. 33232202

[10] Pombubpa N, Pietrasiak N, De Ley P, Stajich JE. 2020. Insights into dryland biocrust microbiome: geography, soil depth and crust type affect biocrust microbial communities and networks in Mojave Desert, USA. FEMS Microbiol Ecol 96:fiaa125. doi:10.1093/femsec/fiaa125. 32573682 PMC7426032

[11] Smith DP, Peay KG. 2014. Sequence depth, not PCR replication, improves ecological inference from next generation DNA sequencing. PLoS One 9:e90234. doi:10.1371/journal.pone.0090234. 24587293 PMC3938664

[12] Caporaso JG, Kuczynski J, Stombaugh J, Bittinger K, Bushman FD, Costello EK, Fierer N, Peña AG, Goodrich JK, Gordon JI, Huttley GA, Kelley ST, Knights D, Koenig JE, Ley RE, Lozupone CA, McDonald D, Muegge BD, Pirrung M, Reeder J, Sevinsky JR, Turnbaugh PJ, Walters WA, Widmann J, Yatsunenko T, Zaneveld J, Knight R. 2010. QIIME allows analysis of high-throughput community sequencing data. Nat Methods 7:335–336. doi:10.1038/nmeth.f.303. 20383131 PMC3156573

[13] Palmer JM, Jusino MA, Banik MT, Lindner DL. 2018. Non-biological synthetic spike-in controls and the AMPtk software pipeline improve mycobiome data. PeerJ 6:e4925. doi:10.7717/peerj.4925. 29868296 PMC5978393

[14] Callahan BJ, McMurdie PJ, Rosen MJ, Han AW, Johnson AJA, Holmes SP. 2016. DADA2: high-resolution sample inference from Illumina amplicon data. Nat Methods 13:581–583. doi:10.1038/nmeth.3869. 27214047 PMC4927377

[15] Lindahl BD, Nilsson RH, Tedersoo L, Abarenkov K, Carlsen T, Kjøller R, Kõljalg U, Pennanen T, Rosendahl S, Stenlid J, Kauserud H. 2013. Fungal community analysis by high‐throughput sequencing of amplified markers—a user’s guide. New Phytol 199:288–299. doi:10.1111/nph.12243. 23534863 PMC3712477

[16] Edgar RC, Flyvbjerg H. 2015. Error filtering, pair assembly and error correction for next-generation sequencing reads. Bioinformatics 31:3476–3482. doi:10.1093/bioinformatics/btv401. 26139637

